# Self-Powered Acceleration Sensor for Distance Prediction via Triboelectrification

**DOI:** 10.3390/s24124021

**Published:** 2024-06-20

**Authors:** Zhengbing Ding, Dinh Cong Nguyen, Hakjeong Kim, Xing Wang, Kyungwho Choi, Jihae Lee, Dukhyun Choi

**Affiliations:** 1 School of Mechanical Engineering, Sungkyunkwan University, Suwon 16419, Republic of Korea; zbding@g.skku.edu (Z.D.); congnd@skku.edu (D.C.N.); versatilehak@nate.com (H.K.); xingwang@g.skku.edu (X.W.); kw.choi@skku.edu (K.C.); 2 Department of Golf Industry, Kyung Hee University, Yongin 17104, Republic of Korea; 3 Department of Future Energy Engineering, Sungkyunkwan University, Suwon 16419, Republic of Korea

**Keywords:** triboelectric nanogenerator, self powered, acceleration sensor, distance prediction

## Abstract

Accurately predicting the distance an object will travel to its destination is very important in various sports. Acceleration sensors as a means of real-time monitoring are gaining increasing attention in sports. Due to the low energy output and power density of Triboelectric Nanogenerators (TENGs), recent efforts have focused on developing various acceleration sensors. However, these sensors suffer from significant drawbacks, including large size, high complexity, high power input requirements, and high cost. Here, we described a portable and cost-effective real-time refreshable strategy design comprising a series of individually addressable and controllable units based on TENGs embedded in a flexible substrate. This results in a highly sensitive, low-cost, and self-powered acceleration sensor. Putting, which accounts for nearly half of all strokes played, is obviously an important component of the golf game. The developed acceleration sensor has an accuracy controlled within 5%. The initial velocity and acceleration of the forward movement of a rolling golf ball after it is hit by a putter can be displayed, and the stopping distance is quickly calculated and predicted in about 7 s. This research demonstrates the application of the portable TENG-based acceleration sensor while paving the way for designing portable, cost-effective, scalable, and harmless ubiquitous self-powered acceleration sensors.

## 1. Introduction

The concept of intelligent and portable products has become deeply entrenched in the public imagination [1,2], signifying that, concomitant with the rapid growth of the internet, there is an accelerated demand for integrated systems, intelligence, and miniaturization, hastening research into multifunctional sensors [3,4,5]. Moreover, as the standard of living rises, so does public interest in health and sports activities, leading to a greater focus on the effectiveness of monitoring both the methods and outcomes of physical exercise. In particular, following the COVID-19 pandemic, the pace of research into multifunctional sensors has quickened due to the heightened demand for system integration, intelligent capability, and compact design [6,7,8]. Collecting real-time data depends on distributed sensors, leveraging many detection mechanisms including optical [2,4], capacitive [9,10], resistive [11,12,13], geomagnetic [14,15,16], chemical [17,18], and thermal sensitivity [19,20,21]. In the arena of intelligent sports, various sensor technologies have been reported that are noted for their high sensitivity and diverse functionalities. However, a standard limitation is their dependency on external power sources, necessitating continuous replacement. Even as technological progress has reduced power consumption for each sensor, the number of such units in sports applications might be significant. Given the limited lifespan, high replacement costs, and environmental pollution concerns associated with battery use, developing a maintenance-free and sustainable sensor technology for monitoring motion data during sporting activities is imperative [22,23,24,25].

Extensive research has shown that, due to the combined effects of contact electrification and electrostatic induction, TENGs are an effective method for converting environmental mechanical energy into electrical energy [26,27]. TENGs possess numerous advantages, such as high efficiency, straightforward architecture, low cost, small size, a wide selection of materials, easy scalability, and a self-powering system that operates without needing an external power source [28,29,30,31,32]. TENGs, based on four fundamental operational modes, have been demonstrated and extensively applied to a variety of forms of mechanical energy, including wind [33,34,35], hydro [36,37,38,39], and kinetic energy [40,41,42], providing a sustainable power source for electronic devices [43] that consume low energy.

In this study, we present a self-powered acceleration sensor based on TENGs to predict the distance a golf ball travels before reaching the hole. We systematically explored the performance of the acceleration and distance monitoring sensor from both theoretical and experimental perspectives. Attaching the sensor to the bottom of a golf practice mat results in the generation of varying voltage signals when an object moves over the hidden sensors. The object’s acceleration and the predicted distance to its stopping point are automatically calculated based on the time difference between these voltage signals. The sensors utilized a slim film design with a thickness of 1.5 μm and a weight of 4.316 g, guaranteeing that discrepancies in the distance predicted through the waveform signal stay within a 5% margin of the actual distance achieved by the object. Additionally, the sensors we have developed are judged based on the time difference in the voltage signal waveform. Even when the environment changes from sunny to rainy, or when the air humidity increases, the surface charge density of the TENG decreases but the waveform characteristics of the sensor output remain almost unchanged [44]. This highlights their considerable potential for practical application. According to recent reports, our sensors have the simplest structure, the lightest weight, and the highest detection range, as well as good integration and excellent stability. Furthermore, the sensor has been experimentally proven to predict the final putt distance of a golf ball, detecting the acceleration and rolling distance, thereby aiding athletes in adjusting their force during golf play. This research further expands the commercial application of TENGs as self-powered sensors in the sports domain.

## 2. Materials and Methods

### 2.1. The Fabrication of TENG

The acceleration sensor consists of carbon film (thickness 1.5 μm), aluminum tape, and conductive wires. It is embedded beneath the golf practice mat, reinforced with ultra-thin film material for charge transfer. When an object rolls in a straight line over the mat containing the sensor, it generates a voltage signal that is transmitted to the device.

### 2.2. Acceleration Measurement and Distance Prediction

The two times $t_{1}$ and $t_{2}$ generated when the object passes over the sensor-equipped mat are crucial in the tests of acceleration measurement and velocity prediction. By connecting the voltage signals generated by the object’s rolling to a computer program and analyzing them, the times $t_{1}$ and $t_{2}$ can be determined. Then, based on the initial kinetic energy, it is possible to calculate the object’s acceleration during motion and predict the distance of straight-line rolling.

## 3. Results and Discussion

**Figure 1:**
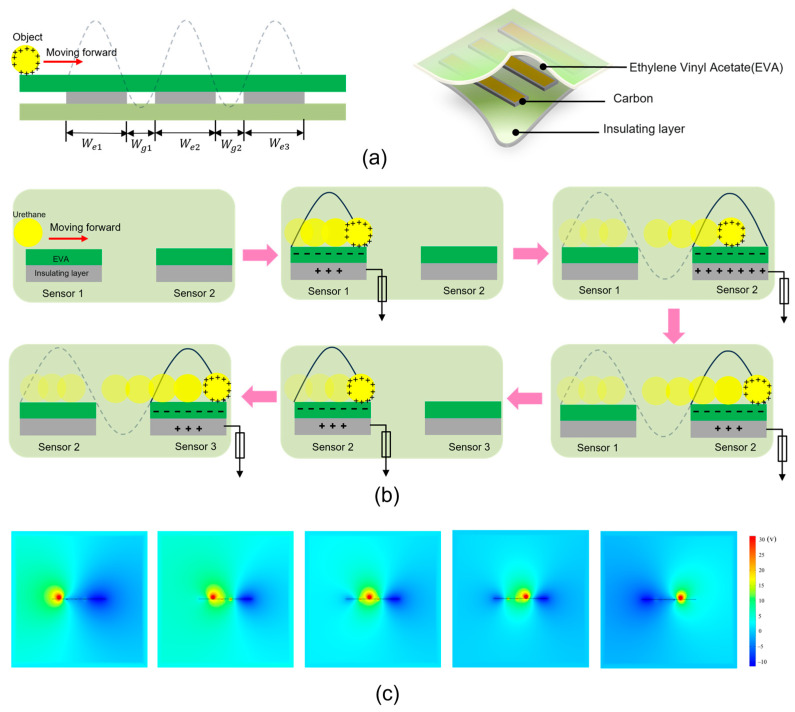
Structural design and working principle of the acceleration sensors. (**a**) Structural diagram of the acceleration sensor. (**b**) When an object moves horizontally above the sensor, electrostatic induction occurs between the object and the three sensors, charge transfer takes place between the object and the sensor material, and the corresponding voltage signal is shown on the oscilloscope. (**c**) Simulation of the potential difference between the object and the sensor material during the contact electrification phase using COMSOL Multiphysics 5.6.

Figure 1a shows the schematic of the main structure of an acceleration sensor based on a single-electrode TENG. The three sensors are embedded equidistantly beneath a mat composed of carbon material and cut to equal size using a laser cutter (C30 Laser Machine, CORYART Company, Anyang, Korea). The three sensors are adhered equidistantly under the pad with leads, as shown in Figure 1a on the left. Note that the signal generation method of the TENG here is a single-electrode mode, so one end of the sensor needs to be in contact with the ground. Since the environment is indoors, an insulating layer is added below the sensor to ensure the efficient transmission of electrons. Figure 1a on the right shows a structural diagram of the sensor, with three sensors equidistantly embedded in the mats and insulating materials. The mat material consists of ethylene vinyl acetate (EVA), the sensor is made of carbon material, and the bottom insulation material is plastic film. The carbon material of the accelerometer produced is easy to obtain, has a rectangular structure of 210 mm length × 60 mm width, and weighs 1.407 g.

The proposed acceleration sensor is designed by embedding multiple sensor units into a flexible substrate according to different needs. Figure 1b shows the working principle. In the initial state, when the object is in contact with the mat, it is located above the sensor electrodes. Electrostatic induction due to the difference in electron affinity between the object and surface causes electrons to transfer from the object to the carbon surface of the sensor, resulting in a negative charge on the carbon film and a positive charge on the surface film of the object. Triboelectric charges cannot be conducted or neutralized for some time. At this stage, the positive tribocharges are fully compensated by the opposite ones, so there is no electrical output produced on the electrode. Once the target ball moves forward and rubs against the film below, the equilibration of the electric field is broken. To balance the potential generated by the system, the potential difference initiates a flow of electrons from the carbon electrode to the ground to balance the potential until a new electrical balance is established (when the object ball rolls to the far right of sensor 1). In a similar manner, the object rolls past sensors 2 and 3. COMSOL simulation shows the potential distribution between two adjacent electrodes on a two-dimensional plane under different conditions. Obviously, the change in the spatial position of the charged object relative to the carbon sensor results in a time-varying spatial potential distribution, thereby generating a potential difference. This drives current to flow in the external circuit, as shown in Figure 1c and the Supporting Movie S1.

Material selection plays a critical role in the structural design and electrical output of sensors based on TENGs. In this work, the object is a golf ball with a urethane surface material, which is known to carry a positive charge according to the triboelectric series. Therefore, for the triboelectric layer, we selected a well-performing carbon film. As the object rolls over the sensor, it forms a corresponding voltage waveform signal based on the amount of charge transferred. Here, the three sensors will generate three corresponding voltage waveform signals. The time it takes for the object to move from the peak of the first voltage to the peak of the second voltage is $t_{1}$, and the time it takes to move from the peak of the second voltage to the peak of the third voltage is $t_{2}$. The distance from the first peak to the second peak is equal to the distance from the second peak to the third peak. From this, the following formula can be derived:

(1) $$\left\{\begin{array}{c} d=v_{0}t_{1}+\frac{1}{2}a{t_{1}}^{2}\\ 2d=v_{0}\left(t_{1}+t_{2}\right)+\frac{1}{2}{a\left(t_{1}+t_{2}\right)}^{2} \end{array}\right.$$

In the above equation, $v_{0}$ represents the initial velocity of the ball. By applying the distance-acceleration relationship, the formula for d and 2d can be derived, thereby obtaining the acceleration a.

The acceleration a is known, and can be calculated by the formula:

(2) $$v_{t}=v_{1}+at$$

Equation (3) can then be obtained:

(3) $$t=\frac{v_{t}-v_{1}}{a}=\frac{\left|-v_{1}\right|}{\left|a\right|}$$

**Figure 2:**
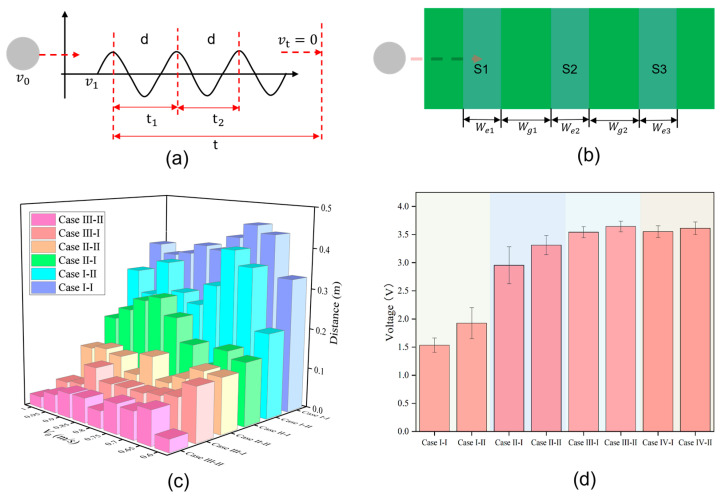
Measurements and performance analysis of the acceleration sensors. (**a**) Calculation of the acceleration and distance based on the voltage signals generated by TENGs. (**b**) The object passing over sensors of different widths $w_{e}$ and space $w_{g}$. (**c**) Errors between the theoretical distance and the actual measured distance for different sensors at various speeds. (**d**) The maximum voltage generated by the object when passing over different sensors.

Finally, the distance *S* from the ball rolling to the stopping position can be obtained, and is shown in Figure 2a as follows:

(4) $$S=v_{1}t+\frac{1}{2}at^{2}$$

The 2D top view in Figure 2b displays the ball passing over sensors with varying widths $w_{e}$ and spacings $w_{g}$. Table 1 lists the different conditions of the sensors. Sensors with different widths at the same spacing or the same sensor at different spacings produce different voltage waveform signals. Error comparison of the theoretical and actual sensor values under different conditions in Figure 2c shows that as the width of sensors or their spacing increases, the error between the calculated and actual measured values of the object rolling to the stop position decreases. More interestingly, as the width of the sensors or their spacing increases, the voltage generated by the object rolling past the sensors gradually increases and then stabilizes, eventually maintaining around 3.5 V, as shown in Figure 2d.

**Figure 3:**
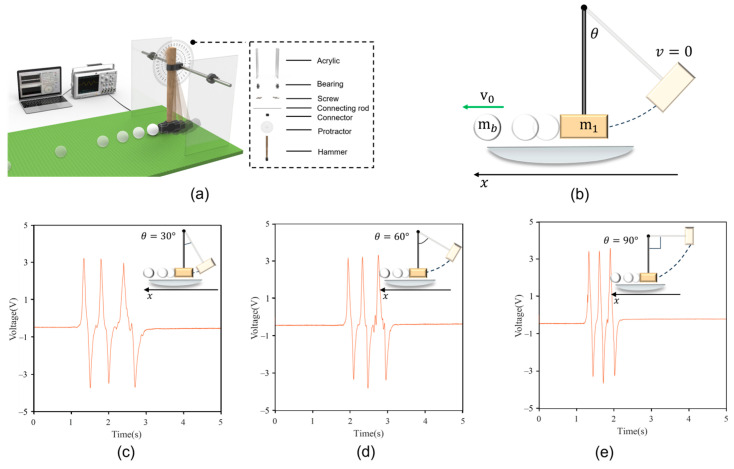
Experimental platform and response characteristics. (**a**) Schematic of the ball motion test under different initial kinetic energies. (**b**) The object acquires different initial kinetic energies by falling through different angles. (**c**–**e**) Graphs of the Voltage−Time relationship of the object rolling forward across the mat after gaining kinetic energy from the hammer falling from angles of 30, 60, and 90°, respectively, as shown in each inset.

According to the explanation in Figure 2, time is the crucial parameter when the ball passes through the three sensors. To verify the working principle of the sensors, we designed and constructed a device that allows the object to roll over a mat with different initial kinetic energies. This device mainly consists of an acrylic board, roller bearings, protractor boards, and a hammer, as shown in Figure 3a. The hammer is raised to a certain angle and then falls to hit the object, imparting initial kinetic energy for the object to move forward in a straight line. We selected bearings to drive the hammer’s vertical motion, significantly reducing the friction during the hammer’s movement, so the friction is negligible. The object rolls horizontally forward and passes sequentially through three sensors located beneath the mat when it gains initial kinetic energy. Voltage waveforms with three peaks and troughs can be clearly seen on the oscilloscope. The voltage signal can also be transmitted to a laptop. Our developed program can automatically read the voltage wave signal and quickly analyze and calculate the acceleration, velocity, and predicted distance of the object. The hammer is raised to a certain angle *θ*, and drops from a fixed point to generate kinetic energy for the object, causing it to move horizontally, as shown in Figure 2b. The kinetic energy generated by the hammer dropping from different angles can be calculated using a formula. Here, we have derived the kinetic energy generated by the hammer’s movement, initial velocity, and the linear distance the ball can roll after being struck by the hammer. The angle between the hammer and the plumb bob is θ, and the radius of the hammer’s circular path is r. Assuming the center of mass of the hammer is at the vertex of the circle, the gravitational potential energy of the hammer when it is pulled up is $E_{h}=m_{h}gr(1-cos\theta )$, where $m_{h}$ is the weight of the hammer.

The assumption is made that there is no energy loss during the hammer’s fall. Then, all the gravitational potential energy of the hammer when it reaches the lowest point is converted into kinetic energy, and the speed at this time can be obtained:

(5) $$E_{h}=m_{h}gr\left(1-\cos\theta \right)=\frac{m_{h}{v_{0}}^{2}}{2}$$

Equation (6) can be thus be obtained:

(6) $$v_{0}=\sqrt{2gr(1-\cos\theta )}$$

Then, assuming that the collision between the hammer and the golf ball satisfies the conservation of momentum, the initial speed of the golf ball after the collision can be obtained:

(7) $$m_{h}v_{0}=m_{h}\sqrt{2gr\left(1-\cos\theta \right)}=m_{b}v_{b}$$

where

(8) $$v_{b}=\frac{m_{h}}{m_{b}}\sqrt{2gr(1-\cos\theta )}$$

where $m_{b}$ is the mass of the golf ball, and $v_{b}$ is the initial speed of the golf ball.

Assuming that the friction force on the rolling golf ball is constant, the moving distance of the golf ball can be obtained:

(9) $$E_{b}=m_{b}{v_{b}}^{2}/2=\frac{{m_{h}}^{2}}{m_{b}}gr(1-\cos\theta )=f\cdot S=\mu m_{b}gS=E_{f}$$

(10) $$S=\frac{1}{\mu }\cdot \frac{{m_{h}}^{2}}{{m_{b}}^{2}}r(1-\cos\theta )$$

where f is the friction force, μ is the friction coefficient, and S is the moving distance. Here, the air resistance is very small compared to the friction and can be ignored.

The hammer was raised to 30, 60, and 90°, respectively, generating three kinetic energies of $E_{1}$, $E_{2}$*,* and $E_{3}$. The object was hit by the three different kinetic energies of the hammer and rolled forward at high speed. The object rolled past the three sensors and obtained three different voltage signal waveforms. We selected the sensor with a width of 60 mm and spacing of 400 mm. Figure 3c–e show the voltage signal waveforms obtained by the object passing through the sensor in the three cases. Interestingly, because the sensor is fixed, the maximum voltage of the voltage waveform can reach about 3.5 V. The time intervals $t_{1}$ and $t_{2}$ of the three voltage signals generated can be clearly seen to differ under the different initial kinetic energies. As the kinetic energy increases, the interval between $t_{1}$ and $t_{2}$ shortens.

To evaluate the performance of the acceleration sensor, we selected sensors of the same length but different widths and measured them under different gravitational potential energy conditions. This process entailed monitoring the initial velocity and acceleration of a rolling object. The sensors were strategically positioned beneath the mat, maintaining a consistent 200 mm gap between the various sensor types. By dropping a hammer from distinct angles, we imparted different initial kinetic energies to the ball, designated as $E_{1}$, $E_{2}$, and $E_{3}$, corresponding to angles of 30, 60, and 90°, respectively (Figure 3c–e, insets). The acceleration and velocity of the object rolling forward after the hammer hit were recorded.

**Figure 4:**
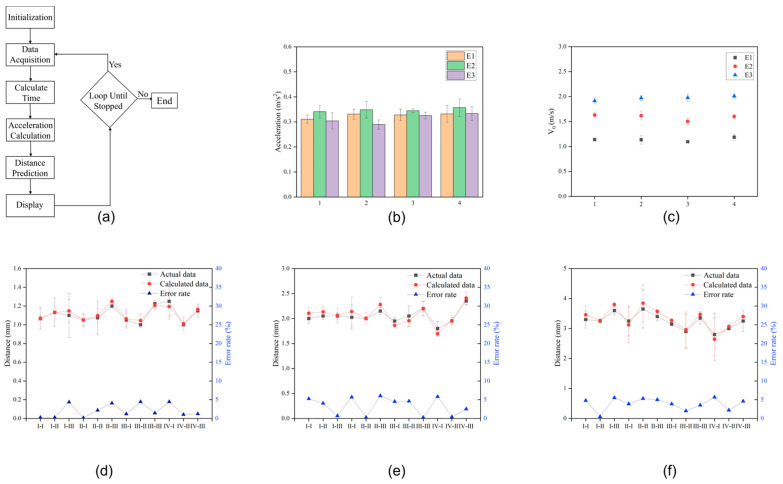
Comparison of different performances of the TENG-based acceleration sensors. (**a**) Algorithmic block diagram of acceleration sensors. (**b**,**c**) The acceleration and speed display, respectively, of the object passing through different types of sensors under different initial kinetic energies. (**d**–**f**) The predicted distance and actual distance that the object can roll under different initial kinetic energies and the error involved, respectively.

According to the mechanical robust sensing mechanism, accurately identifying the starting and ending points of the output waveform is the key to realizing the distance prediction of the acceleration sensor. To accurately obtain the two time differences ($t_{1}$ and $t_{2}$) between the peak values of the waveform signal, a set of signal processing and feature recognition algorithms is proposed for the sensor (Figure 4a). Figure 4b, c shows the acceleration and velocity of the object when driven by the three amounts of kinetic energy and passing through acceleration sensors of widths 60, 80, 100, and 120 mm. Table 2 lists four sensors with different widths and corresponding to different spacings. The voltage waveform signals generated by the sensors in different situations are different. As the width of the acceleration sensor increases or the initial kinetic energy increases, the acceleration of the object during rolling can be observed to remain roughly constant at about 0.3 m/s^2^. It can also be clearly seen that as the initial kinetic energy of an object increases, the speed of the object increases, but under the same initial kinetic energy conditions, the initial speed of the object is basically the same. Experiments show that the acceleration of the object in this study has nothing to do with the initial kinetic energy, and the amount of kinetic energy affects the velocity $v_{0}$ of the object. 

The distance after the object rolls past the acceleration sensor can be automatically calculated. Three different kinetic energies ($E_{1}$, $E_{2}$, and $E_{3}$) were used, and after the hammer hit the object, the estimated stopping distance past different sensors from case I–I to case IV–III was recorded and compared with the actual measured distance. In this study, to compare the calculated values of the object’s moving distance with the actual measured values, the calculated values were found to be almost consistent with the actual measured distances of the object’s movement, with an error margin of around 5% (Figure 4d–f). This indicates that the acceleration sensor based on the nanogenerator for frictional electricity demonstrates significant accuracy in distance prediction functionality.

Compared to other rigid-structured acceleration sensors, we further explored the functionalities of sensors based on the nanogenerator for frictional electricity by embedding simple sensor units into a flexible golf practice mat. To demonstrate the capability of the acceleration sensor in predicting distances, we proposed a scenario where the fabricated acceleration sensors predict the travel distance of a golf ball.

**Figure 5:**
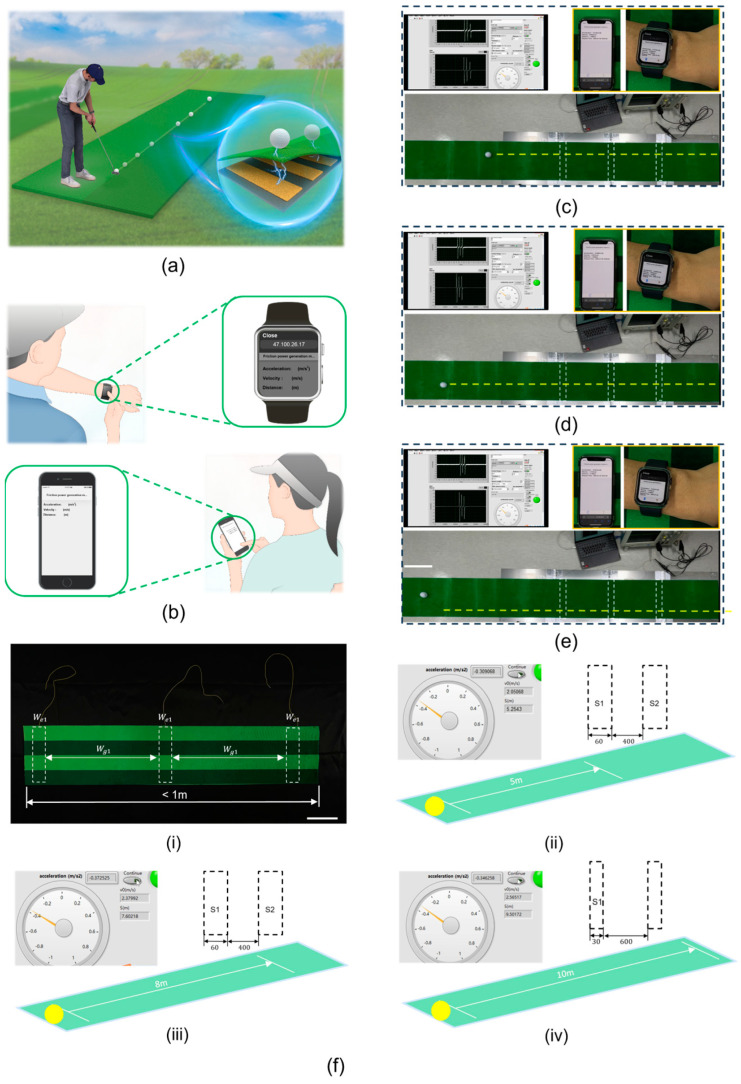
Practical application of the acceleration sensor based on TENG in golf sports. (**a**) Schematic of an athlete hitting a golf ball. (**b**) Schematic showing that the data read by the acceleration sensor can be automatically transmitted to mobile phones and smartwatches. (**c**–**e**) The golf ball passes through the TENG sensor to obtain its actual acceleration, speed, and distance on mobile phones and smartwatches. Scale bar: 20 cm. (**f**,**i**) The fabricated 1 m sensor-containing mat of 60 mm width × 400 mm spacing. Scale bar: 10 cm. (**ii**,**iii**) The sensor in (**i**) can predict a ball rolling to about 5 m and 8 m, respectively. (**iv**) A sensor with a width of 30 mm and a spacing of 600 mm can predict the ball rolling to a distance of about 10 m.

First, Figure 5a illustrates the motion of a golfer hitting the ball into the hole, with an inset showing the principle of TENGs as the golf ball passes over the sensor beneath the mat. As the golf ball rolls forward, passing over the TENG sensor, three distinct voltage waveforms are visible on the oscilloscope. Then, by analyzing the time interval of these voltage waveform signals, the motion acceleration and rolling distance of the golf ball can be calculated and transmitted in real time to smartphones and smartwatches, allowing players or referees to quickly obtain data results, as shown in Figure 5b. Last, tests were conducted on a 3 m long golf practice mat with the golf ball struck with varying force in three scenarios. As depicted in Figure 5c–e, and Supporting Movies S2–S4, the golf ball rolled distances of 1.489, 1.948, and 2.189 m under three different force conditions. The initial velocity, rolling acceleration, and rolling distance of the golf ball were clearly displayed on a computer interface, with smartphones and smartwatches also able to simultaneously receive the same data. We continued to increase the force of hitting a golf ball. The acceleration sensor based on a TENG can predict the distance to about 5 m or even 8 m. As the predicted distance extends, achieving a clear and stable waveform time $t_{1}$ and $t_{2}$, we need to change the size and spacing of the acceleration sensors. When we change the width of the acceleration sensors to 30 mm and the spacing to 600 mm, the acceleration sensors can predict distances of up to about 10 m, as shown in Figure 5f(i–iii). We successfully produced a mat containing a sensor that is less than 1m in length and can predict the linear movement distance of an object up to about 10 m. This length of distance prediction is sufficiently comprehensive for golf swing training before the ball enters the hole. This prototype of a TENG–based embedded acceleration sensor demonstrates the potential of creating sensitive and effective commercial sensors for real-time monitoring and assessment in golf sports.

## 4. Conclusions

In summary, we have integrated a nanogenerator for frictional electricity into the design of a system for monitoring the rolling distance of a golf ball. This system allows for the direct observation of real-time data through electronic devices, representing a wide range of potential applications. By determining the size of the sensors and strategically utilizing different force variations to strike the golf ball, the distance it can roll is calculated based on the changes in the signal of the voltage waveform over time. By choosing materials with opposite charges for the golf ball’s surface, the frictional electricity generated between the two materials creates a voltage signal, calculating both acceleration and distance. With the help of intelligent signal processing algorithms, acceleration calculation, and distance sensing can be achieved based on the characteristics of the sensor voltage signal rather than the amplitude. Compared to previous research, the efficiency of the acceleration sensor based on the nanogenerator for frictional electricity has increased several-fold. The data monitored by the TENG-based acceleration sensor is within an acceptable range, showing its high sensitivity and excellent stability. Specifically, the system response time is no more than 7 s, and the error between the predicted distance and the actual distance is less than 5%. The TENG-based embedded acceleration sensor offers strong independence and flexibility, being adaptable to real-world scenarios with a millimeter-level thickness that further demonstrates the potential for designing self-powered sensor systems. The relationship between the electrical output characteristics and the applied acceleration has been theoretically and experimentally explored.

Additionally, the acceleration sensor can operate under its own power, making it suitable for portable devices without bulky accessories. The ease of material acquisition and fabrication also ensures lower production costs at various scales. Finally, the acceleration sensor is used to monitor golfers’ training and to precisely measure the rolling distance of the golf ball during the final putt. Similarly, acceleration sensors can also be applied to predict distances in sports such as gateball, and can predict the continued move distance of skateboard wheels in skateboarding when descending from heights. This work demonstrates a self-powered acceleration sensor and expands the commercialization and application of TENGs in self-powered sensing systems. Our research thus opens pathways for creating compact, portable, economical, scalable, and harmless ubiquitous self-powered sensing systems.

## Figures and Tables

**Table 1:** Three sensors with the same width, with widths of 20 mm, 40 mm, 60 mm, and 80 mm, respectively, with different spacing between sensors (200 mm, 400 mm, and 600 mm).

Case	$w_{e}$ (mm)	$w_{g}$ (mm)
I–I	20	200
I–II		400
II–I	40	200
II–II		400
III–I	60	200
III–II		400
IV–I	80	200
IV–II		400

**Table 2:** Four sensors with the same width, with widths of 60 mm, 80 mm, 100 mm, and 120 mm, respectively, and with different spacings between sensors (200 mm, 400 mm, and 600 mm).

Case	$w_{e}$ (mm)	$w_{g}$ (mm)
I–I	60	200
I–II		400
I–III		600
II–I	80	200
II–II		400
II–III		600
III–I	100	200
III–II		400
III–III		600
IV–I	120	200
IV–II		400
IV–III		600

## Data Availability

Data are contained within the article.

## Supplementary Materials

The following supporting information can be downloaded at: https://www.mdpi.com/article/10.3390/s24124021/s1. Movie S1. Simulation of voltage changes when a golf ball passes through a practice mat. Movie S2. Demonstration of a golfer hitting a golf ball with less force. Movie S3. Demonstration of a golfer hitting a golf ball with moderate force. Movie S4. Demonstration of a golfer hitting a golf ball with great force. Figure S1. The fabrication process of the acceleration sensors. Figure S2. Schematic diagram of the object ball being hit by the hammer. Figure S3. Display of experimental platform.
