# Supplementary material for: Self-Powered Acceleration Sensor for Distance Prediction via Triboelectrification
Source: Sensors (Basel). 2024 Jun 20;24(12):4021. doi: 10.3390/s24124021 (PMC11209619; doi:10.3390/s24124021)
Supplement: Supplementary file 1 [file sensors-24-04021-s001.zip › Supporting Information.pdf]

sensors

Supporting Information

Self-Powered Acceleration Sensor for Distance Prediction via Triboelectrification

Zhengbing Ding [1], Dinh Cong Nguyen [1], Hakjeong Kim [1], Xing Wang [1], Kyungwho Choi [1], Jihae Lee [2,*] and Dukhyun Choi [1,3,*]

[1] School of Mechanical Engineering, Sungkyunkwan University, Suwon 16419, Republic of Korea
[2] Department of Golf Industry, Kyung Hee University, Yongin 17104, Republic of Korea
[3] Department of Future Energy Engineering, Sungkyunkwan University, Suwon 16419, Republic of Korea
* Correspondence: cheesehead85@khu.ac.kr (J.L.); bred96@skku.edu (D.C.)

The PDF file includes:

1. Triboelectric materials in series following a tendency to lose electrons and to gain electrons.
2. The fabrication process of the acceleration sensors. (Fig.S1)
3. Calculation of kinetic energy E_1, E_2, and E_3 and initial velocity of the object. (Fig.S2)
4. Construction and display of experimental platform. (Fig.S3)

Other Supporting Information for this manuscript includes the following:

4. Simulation of voltage changes when a golf ball passes through a practice mat (Movie S1)
5. Demonstration of a golfer hitting a golf ball with less force (Movie S2)
6. Demonstration of a golfer hitting a golf ball with moderate force (Movie S3)
7. Demonstration of a golfer hitting a golf ball with great force (Movie S4)

1. Table S1 A list that ranks various materials according to their tendency to gain (negative) or lose electrons (positive) in the contact charging process.

**Positive**	Human skin (especially dry)	（Continued）	
	Leather	Hard rubber	
	Glass	Nickel, Copper	
	**Urethane (Polyurethane)**	Brass, Silver	
	Wood	Gold, Platinum	
	Fur	Polyester	
	Silicon	Polyvinyl chloride (PVC)	
	Paper	Silicone	
	Cotton	Teflon	
	Steel	Silicon Rubber	
	(continued)	**Carbon** (graphite, carbon black, carbon fiber)	**Negative**

In this simplified series, Urethane is placed above the neutral point, indicating it tends to lose electrons (becoming positively charged) when in contact with materials listed below it. Carbon is placed towards the negative end of the series, suggesting it has a tendency to gain electrons (becoming negatively charged) when in contact with most other materials above it in the series.

2. The fabrication process of the acceleration sensors.

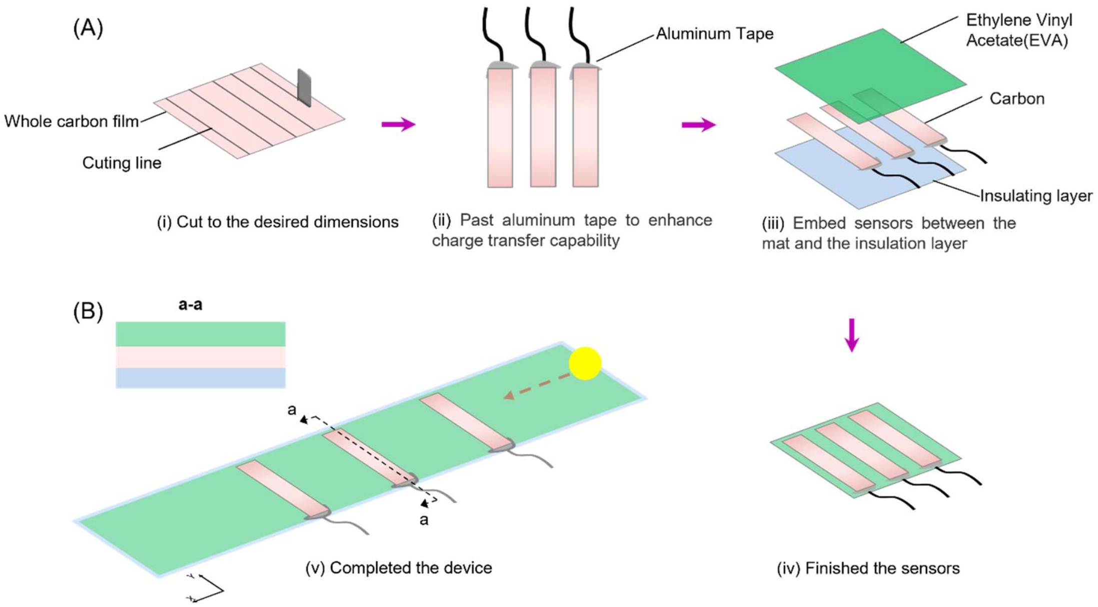

Figure S1. The fabrication process of the acceleration sensors. (A) The step-by-step fabrication process of the sensors is illustrated through (i)~(iii). The complete sensors and device are shown in (iv) and (v). (B) Schematics of the cross-section of the acceleration sensors.

Cut sensors from the carbon film according to the required dimensions. Then, to enhance the charge transfer capability between materials, pasted an appropriate aluminum tape beneath the carbon and connect it to wires for later use. Next, affixed the carbon sensor beneath an EVA material golf practice mat, and finally, layed an insulation layer at the very bottom to prevent interference with the charge transfer process. The entire manufacturing process is illustrated in Figure S1, from (i) to (iv). Finally, S1 B demonstrates the application of the accelerometer in golf swings. The inset images are actual photographs.

3. Calculation of kinetic energy E_1, E_2, and E_3 and initial velocity of the object.

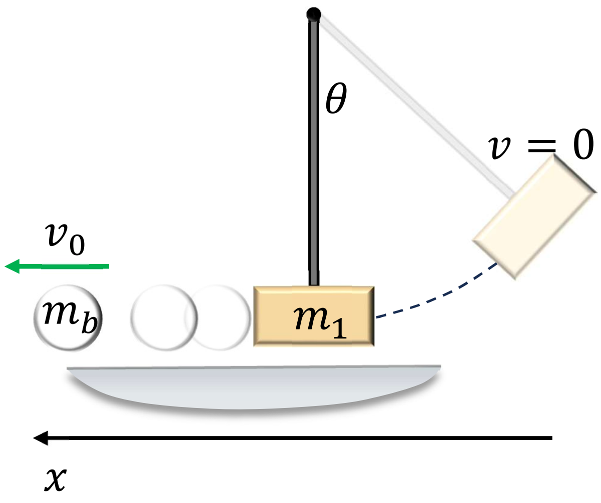

Figure S2. Schematic diagram of the object ball being hit by the hammer.

The gravitational potential energy of the hammer when it is pulled up is $E_h = m_1 gr(1 - cos\theta)$, where $m_1 = 81.5g$ is the weight of the hammer. (And $r = 21cm$)
Here, assume that there is no energy loss during the hammer's fall. Then all the gravitational potential energy of the hammer when it reaches the lowest point is converted into kinetic energy, and the speed at this time can be obtained:

$$E_h = m_1 gr(1 - cos\,\theta) = \frac{m_1 {v_0}^2}{2} = E_m \tag{S1}$$

So, we can get

$$v_0 = \sqrt{2gr(1 - cos\,\theta)} \tag{S2}$$

Then, assume that the collision between the hammer and the golf ball satisfies the conservation of momentum. Then, we can get the initial speed of the golf ball after the collision:

$$m_1 v_0 = m_1 \sqrt{2gr(1 - cos\,\theta)} = m_b v_b \tag{S3}$$

Finally,

$$v_b = \frac{m_1}{m_b} \sqrt{2gr(1 - cos\,\theta)} \tag{S4}$$

(where $m_b = 46g$ is the mass of the golf ball, and v_b is the initial speed of the golf ball).
We can calculate respectively through formula S1: $E_1 = 0.0025J$, $E_2 = 0.084J$, $E_3 = 0.168J$. And we can also calculate the initial speed of the object ball according to formulas S2, S3, and S4. Finally, we got the initial velocities under three kinetic energies: $v_{b_1} = 1.32m/s$, $v_{b_2} = 2.54m/s$, $v_{b_3} = 3.59m/s$.

4. Construction and display of experimental platform.

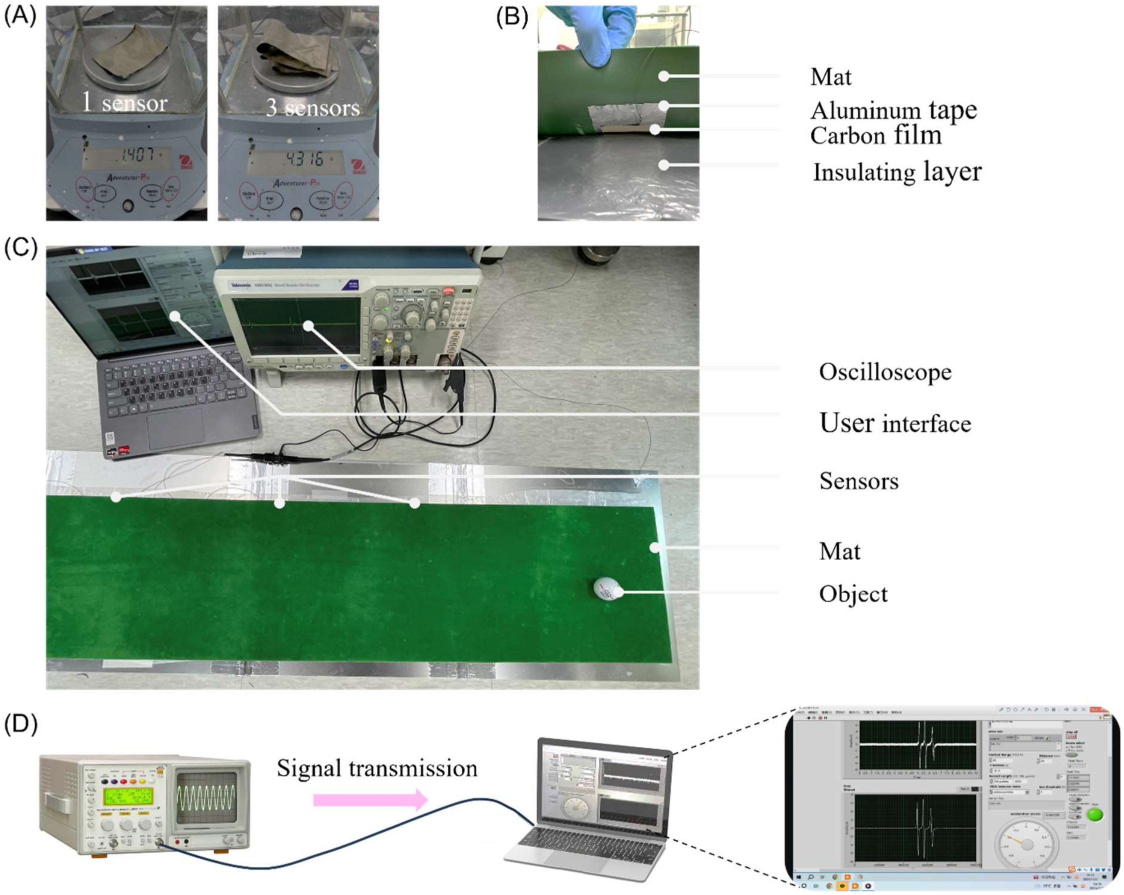

Figure S3. Display of experimental platform. (A) Weight display of Carbon film. (B) Sensor installation structure. (C) Acceleration sensor data display and sorting. (D) Real-time monitoring data.

The experimental results show that the material we selected is lightweight and deformable, and it does not affect training at all when embedded under the golf practice blanket. The data received by the oscilloscope can be transferred to the computer in real-time for automatic calculation and display of the object's acceleration, initial velocity, and predicted distance results with high accuracy.
