# Supplementary figures and images for: Self-Powered Acceleration Sensor for Distance Prediction via Triboelectrification
Source: Sensors (Basel). 2024 Jun 20;24(12):4021. doi: 10.3390/s24124021 (PMC11209619; doi:10.3390/s24124021)

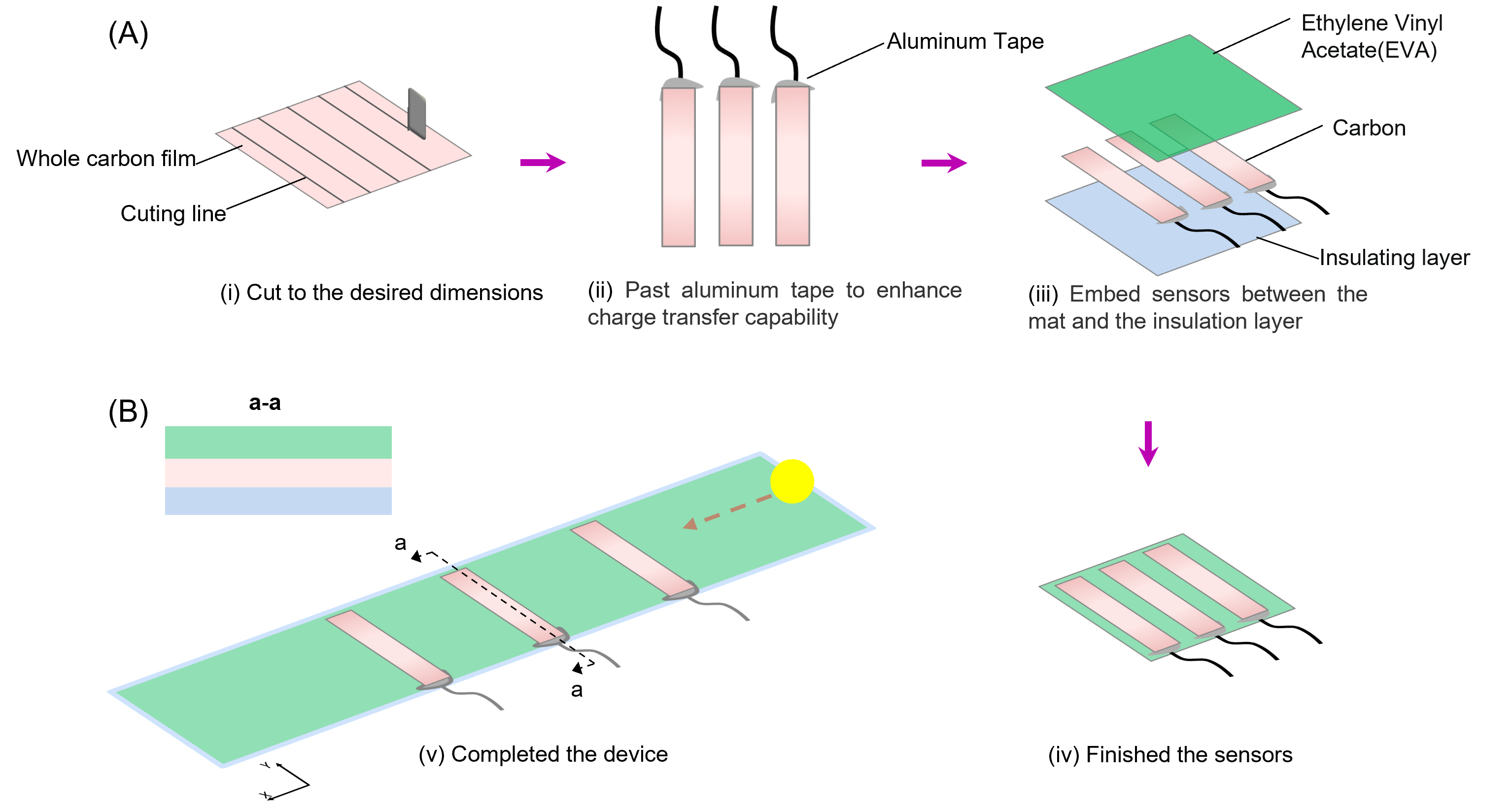

Supplement: Supplementary file 1 [file sensors-24-04021-s001.zip › Figure S1.tif]

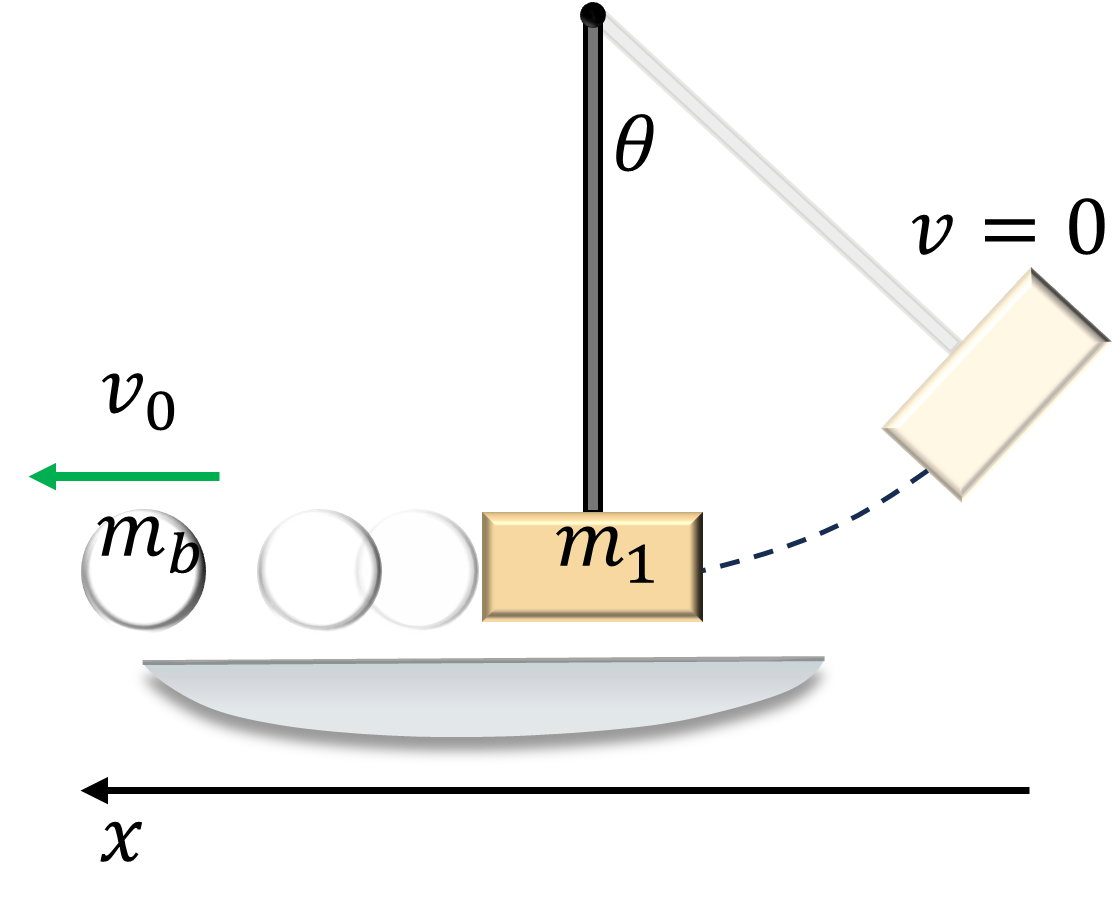

Supplement: Supplementary file 1 [file sensors-24-04021-s001.zip › Figure S2.tif]

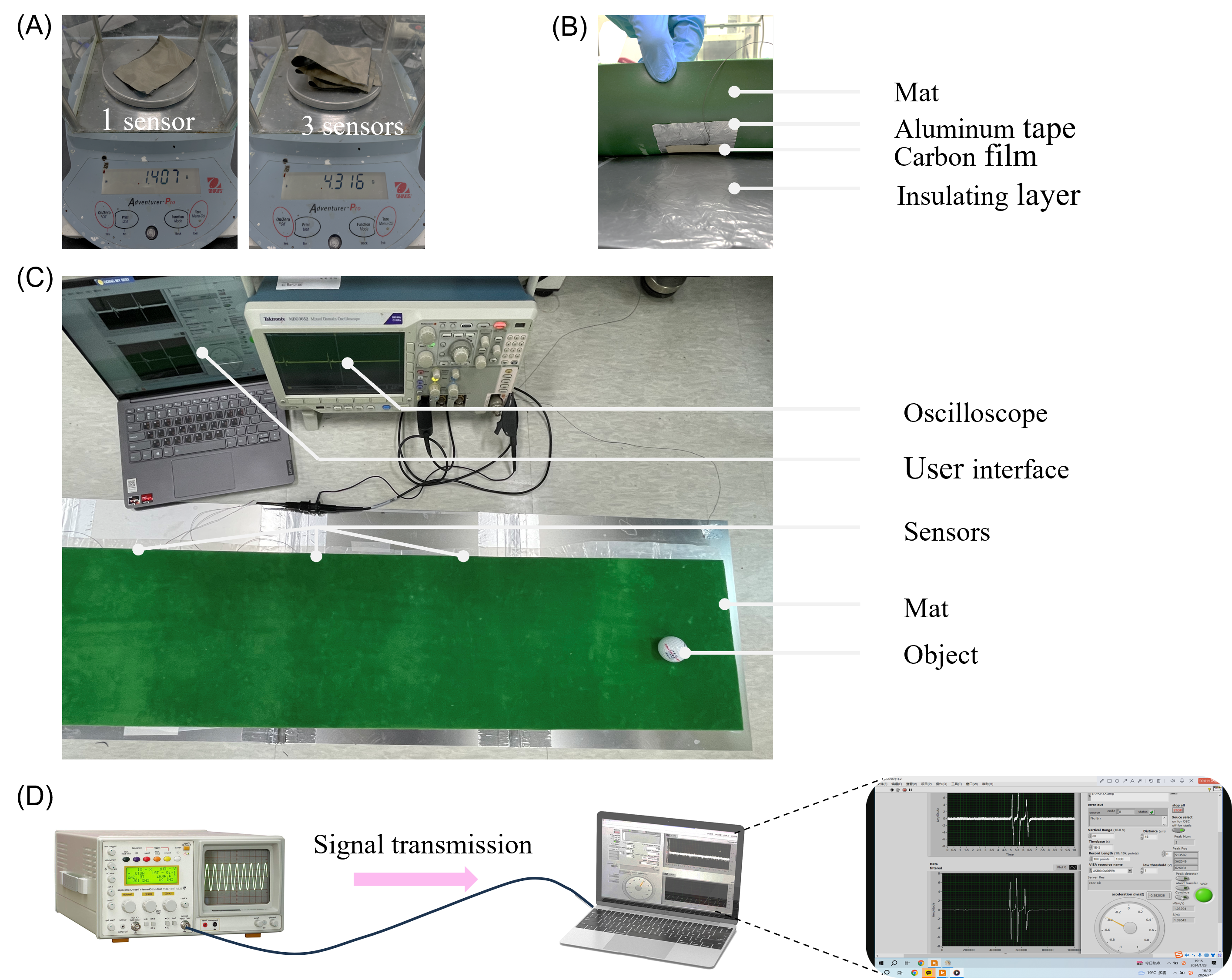

Supplement: Supplementary file 1 [file sensors-24-04021-s001.zip › Figure S3.tif]
